# Architecting functionalized carbon microtube/carrollite nanocomposite demonstrating significant microwave characteristics

**DOI:** 10.1038/s41598-021-91370-5

**Published:** 2021-06-07

**Authors:** Reza Peymanfar, Elnaz Selseleh-Zakerin, Ali Ahmadi, Seyed Hassan Tavassoli

**Affiliations:** 1 Laser and Plasma Research Institute, Shahid Beheshti University, Tehran, 1983969411 Iran; 2 Department of Chemical Engineering, Energy Institute of Higher Education, Saveh, Iran

**Keywords:** Materials chemistry, Electronic properties and materials, Nanoparticles, Structural properties, Composites

## Abstract

Biomass-derived materials have recently received considerable attention as lightweight, low-cost, and green microwave absorbers. On the other hand, sulfide nanostructures due to their narrow band gaps have demonstrated significant microwave characteristics. In this research, carbon microtubes were fabricated using a biowaste and then functionalized by a novel complementary solvothermal and sonochemistry method. The functionalized carbon microtubes (FCMT) were ornamented by CuCo_2_S_4_ nanoparticles as a novel spinel sulfide microwave absorber. The prepared structures illustrated narrow energy band gap and deposition of the sulfide structures augmented the polarizability, desirable for dielectric loss and microwave attenuation. Eventually, the architected structures were blended by polyacrylonitrile (PAN) to estimate their microwave absorbing and antibacterial characteristics. The antibacterial properties against Gram-negative *Escherichia coli* (*E. coli*) and Gram-positive *Staphylococcus aureus* (*S. aureus*) were scrupulously assessed. Noteworthy, the maximum reflection loss (RL) of the CuCo_2_S_4_/PAN with a thickness of 1.75 mm was 61.88 dB at 11.60 GHz, while the architected FCMT/PAN composite gained a broadband efficient bandwidth as wide as 7.91 GHz (RL > 10 dB) and 3.25 GHz (RL > 20 dB) with a thickness of 2.00 mm. More significantly, FCMT/CuCo_2_S_4_/PAN demonstrated an efficient bandwidth of 2.04 GHz (RL > 20 dB) with only 1.75 mm in thickness. Interestingly, FCMT/CuCo_2_S_4_/PAN and CuCo_2_S_4_/PAN composites demonstrated an electromagnetic interference shielding efficiency of more than 90 and 97% at the entire x and ku-band frequencies, respectively.

## Introduction

The benefits of microwave absorbing materials are clear to anyone caring about his/her health against the harmful electromagnetic waves, emitted from the electronic devices surrounding us in our inescapable mechanical life. The carcinogenicity, reproductive toxicity, genotoxicity, brain tissue injury, neurological damage, and other health hazards associated with microwave have been reported, originated from the human exposure to radiofrequency radiation, known as non-ionizing radiation (30 kHz–300 GHz), which threaten human and any living species^1–6^. Accordingly, microwave absorbing materials have been the hotspot owing to their significant importance in the healthcare, industrial, and military fields. The fabricated microwave absorbing materials can essentially promote our health by loading them into textiles, building materials, and dyes, protecting us against harmful radiations. One of the essential aspects to take into account is the biocompatibility of the employed materials in microwave absorbers, diminishing secondary health damages^7,8^. The achieved results manifest that the size of carbon-based materials and their functional groups are the crucial factors influencing their biocompatibility^9,10^. Moreover, CuCo_2_S_4_, PAN, and carbon-based materials have illustrated the widespread biomedical applications and proper biocompatibility^11–16^. Noticeably, combating bacterial contamination using nanostructures as the hotspot has attracted widespread interest all over the globe. Interestingly, the antibacterial activities of the nanostructures containing Cu, Co, and S elements as well as carbon-based structures have been enormously investigated^17–21^. It is well known that the permeability and permittivity of structures are the crucial factors bringing microwave absorption, given by the transmission line theory. The dielectric feature, conductive loss, and electron hopping pave the way for the permittivity of absorbers. The chemical functional groups, crystal dislocations, and defects alongside the morphology play key roles in promoting the dielectric characteristics^22–29^. Over the past decades, biomass-derived materials due to renewable, eco-friendly, and abundant resources have attracted a great deal of attention as ideal candidates in energy production, conversion, and storage as well as supercapacitors, CO_2_ capture, biogas production, and so on^30–33^. Noteworthy, biomass-derived materials have emerged as light-weight, low-cost, and green microwave absorbers, which their fascinating microwave characteristics are originated from their dielectric and conductive properties^29,34^. Nowadays, apart from carbon-based structures, various structures including transition metal carbides and nitrides (MXenes), metal–organic framework (MOF)-derived materials, nanostructured metals and oxides, as well as other conductive polymers comprising polyaniline, polypyrrole, polythiophene, and polydopamine were enormously used as microwave absorbing materials^35–44^. It should be noted that the cobalt-based spinel oxides have exhibited salient microwave absorbing features meanwhile the sulfide nanostructures have recently intrigued a great deal of interest due to their considerable relaxation loss features, generated by their narrow energy band gaps^45–47^. Among them, CuS, FeS_2_, MoS_2_, and WS_2_ as sulfide nanostructures as well as waxberry, eggshell membrane, wood-based, chicken featherfibers, fish skin, rice, and corn stover as biomass materials were applied to fabricate the microwave absorbing materials^27,48–56^. Light-weight and low-cost electromagnetic wave absorbers with high performances based on biomass-derived reduced graphene oxides (rGO) were reported by Cao et al. The results suggest that biomass-rGO show a maximum RL of 51.7 dB and an efficient bandwidth of 13.5 GHz (4.5–18 GHz) at a thickness of 3.25 mm, implying the unique critical role of the microstructure in adjusting the electromagnetic microwave absorption performance^55^. Li et al. have investigated the microwave absorbing properties of porous C@CoFe_2_O_4_ nanocomposites, derived from the eggshell membrane. The hierarchically porous structures, obtained from the eggshell membrane, and the anchored CoFe_2_O_4_ nanoparticles helped that C/CoFe_2_O_4_ nanocomposites perform a favorable electromagnetic absorption capability. The porous C@CoFe_2_O_4_ nanocomposites achieved the maximum RL of 49.6 dB at 9.2 GHz with 30% loading in the paraffin matrix^51^. MoS_2_ nanosheets were prepared at 180 °C by Ji et al. reaching as high as 47.8 dB at 12.8 GHz due to their high electrical conductivity and the polarization effect. It can also be found that MoS_2_ exhibited an efficient electromagnetic wave absorption bandwidth of 5.2 GHz (RL > 10 dB) at thicknesses of 1.9 and 2.0 mm^57^. Kar et al. have architected lightweight, nature-friendly, and low-cost microwave absorbing materials by pyrolyzing the chicken featherfibers at diverse temperatures. The sample pyrolyzed at 1400 °C attained a maximum RL of 44.6 dB and broad efficient bandwidth sharing 52.9% of the entire x-band frequencies with a thickness of 1.68 mm. The achieved results testified that the dipole, defect, and interfacial polarization, as well as impedance matching, multiple reflections, and multiple scattering, are the vital parameters bringing the microwave features^56^. Recently, the size and medium influence on the microwave absorbing, electromagnetic shielding, optical, and magnetic properties of CuCo_2_S_4_ nanostructures were assessed^58^. Diverse morphologies of carbon-based structures including grapheme, flake, sphere, nanotube, fiber, graphene foam, and carbide were applied as microwave absorbing structures^59–64^. In this study, pure and uniform CMTs were prepared by pyrolyzing a biowaste (*Populus euphratica* harvest) as novel raw material and functionalized by an innovative complementary method. Interestingly, carrollite as a novel spinel sulfide microwave absorber was architected and anchored onto FCMT as well as its synergic effects in FCMT/carrollite composite were scrupulously dissected. It is noteworthy that the antibacterial characteristic of the nanocomposites as well as the used PAN as an absorbing medium, improving mechanical properties compared to the conventional wax, develop the practical applications of the tailored composites.

## Materials and methods

### Materials

Cobalt (II) nitrate hexahydrate, copper (II) acetate monohydrate, N, N-dimethylformamide (DMF), ethanol, and nitric acid (65%) were purchased from Merck. Moreover, sodium sulfide hydrate (60.0–62.0%) was obtained from Samchun Chemicals while PAN was supplied from Sigma-Aldrich. Mueller–Hinton agar was purchased from the IBRESCO meanwhile *E. coli* ATCC 25922 and *S. aureus* ATCC 25923 obtained from Darvash Co. were employed to investigate the antibacterial characteristics.

### Experimental steps

#### Preparation of FCMTs by biomass

CMTs were fabricated by pyrolyzing the harvest of *Populus euphratica* at 500 °C in an N_2_ environment for 3 h. The prepared CMTs were functionalized by a novel modified complementary method. Initially, 0.13 g of CMTs was suspended in 15 ml nitric acid using simultaneously an overhead stirrer and ultrasonic bath for 2 h. Subsequently, the oxygen-containing functional groups fully anchored onto CMTs by a solvothermal process for 2 h at 120 °C. The obtained FCMTs were rinsed by deionized water to natural pH and then were dried at 60 °C. The etching treatments performed by sonochemistry elevate defects at grain boundaries while the functionalizing process was fully done through the solvothermal route. The established defects and functional groups generate the diverse polarization relaxation times, ascending the relaxation loss. More significantly, inserting the oxygen-containing functional groups transfer the sp^2^ hybridization of conjugated CMTs to sp^3^ hybridization, enhancing polarizability.

#### Architecting FCMT/CuCo_2_S_4_ nanocomposite

**Figure 1:**
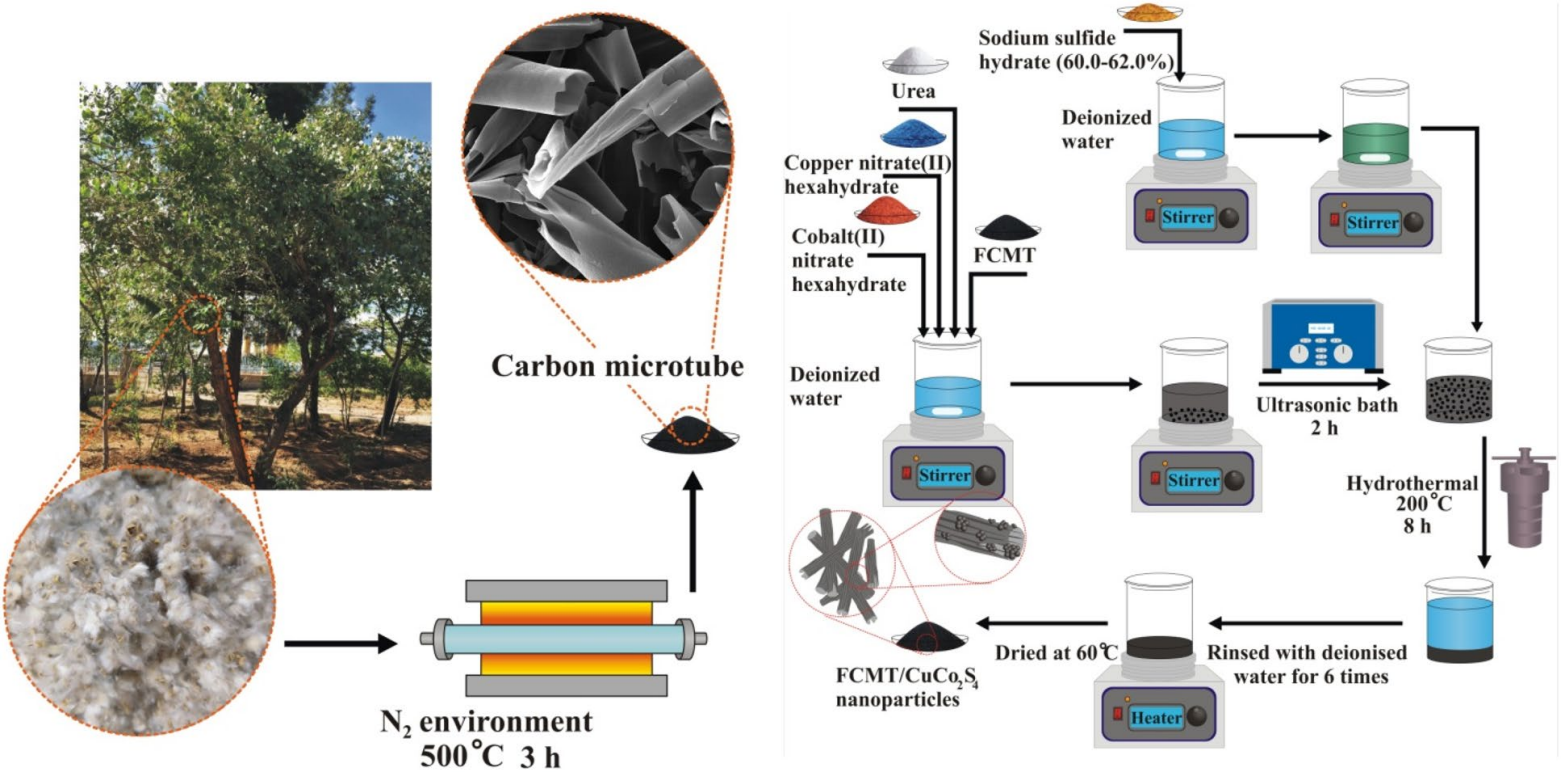
Synthetic route of FCMT/CuCo_2_S_4_ nanocomposite.

Firstly, the copper and cobalt salts in stoichiometric amounts were dissolved in a mixture of deionized water/ethanol = 50% (v/v) and then FCMTs by 10 Wt. % were dispersed in the solution by an ultrasonic bath and overhead stirrer, simultaneously. Afterward, the sodium sulfide in a molar ratio of S^2−^/Cu^2+^ = 6 was separately dissolved in the solvent and added to the aforementioned solution, following that the suspension was treated for 1 h. Next, it was transferred into a p‐polyphenylene lined stainless steel autoclave and annealed for 8 h at 200 °C. The architected nanocomposite was rinsed several times and dried at 60 °C. Eventually, CuCo_2_S_4_ nanoparticles were prepared based on the presented route in the absence of FCMTs. The fabrication procedures of FCMT/CuCo_2_S_4_ nanocomposite have been illustrated in Fig. 1.

#### Preparation of microwave absorbing and antibacterial samples

The microwave absorbing and antibacterial samples were fabricated through a blending process as follows: PAN was dissolved in DMF and then each sample was blended and sonicated there for 30 min. Subsequently, the suspension was molded at 165 °C in the rectangular shapes to measure their microwave features. The filler ratio of CuCo_2_S_4_ and FCMT/CuCo_2_S_4_ nanostructures was guest/guest + host = 50 Wt.% meanwhile it was 5 Wt.% for FCMTs. The chosen amount of FCMTs is in accordance with its ratio in FCMT/CuCo_2_S_4_/PAN nanocomposite.

### Antibacterial assay

A 0.5 McFarland suspension of *E. coli* and *S. aureus* bacteria was spread on an agar culture medium. Then, sterile paper discs (5 mm in diameter) were separately soaked in the saturated solutions of DMF and molded PAN, FCMT/PAN, CuCo_2_S_4_/PAN, or FCMT/CuCo_2_S_4_/PAN, following that the soaked discs were placed on the culture media and incubated at 37 °C for 24 h to evaluate the antibacterial features of the samples.

### Characterization

The chemical species and crystal phases were revealed by Shimadzu 8400 and D8 advance X-ray diffractometer from Bruker, respectively. The optical performance was studied using Shimadzu MPC-2200 while FESEM and TEM images were obtained by Tescan Mira3 and Phillips instruments. IRI Kashan VSM assessed hysteresis loops of the prepared structures, employed at room temperature. The microwave features were provided by an Agilent technology (E8364A).

### FTIR and XRD

**Figure 2:**
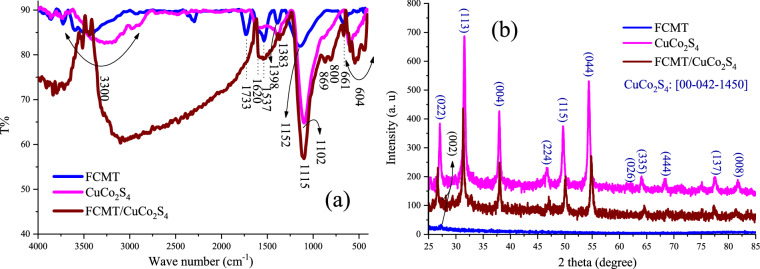
FTIR spectra (**a**) and XRD patterns (**b**) of FCMT, CuCo_2_S_4_, and FCMT/CuCo_2_S_4_ structures.

FTIR spectra and XRD patterns of FCMT, CuCo_2_S_4_, and FCMT/CuCo_2_S_4_ structures have been depicted in Fig. 2. For FCMT spectrum, the assigned peaks at 661 and 1152 cm^−1^ are related to the deformation vibrations of C–H and stretching vibrations of C–O while the observed peak at 1733 cm^−1^ is attributed to the stretching vibrations of C=O functional groups. The shallow band around 3300 cm^−1^ as well as the absorption bands at 1398, 1537, and 1620 cm^−1^ are ascribed to the stretching vibrations of hydroxyl, in-plane and out-of-plane bending vibrations of O–H, as well as symmetric and asymmetric stretching vibrations of C=C in conjugated FCMTs, respectively. Noteworthy, it can be seen that the novel modified method loaded the oxygen-containing functional groups onto CMTs while the chemical structure of CMTs was maintained. For CuCo_2_S_4_ nanoparticles, the shoulder at 604 cm^−1^ refers to the symmetric and asymmetric stretching vibrations of Cu–S and Co–S in the diverse coordinate states^50,65–68^. The notches at 800, 869, 1102, and 1383 cm^−1^ attest to the existing sulphonate, sulfoxide, and sulfones in grain boundaries^65,66,69^. It is found that the water was adsorbed at heterogeneous interfaces of the nanoparticles, suggested by the bumps around 1620 and 3300 cm^−1^. The observed parallel and overlapped peaks in the spectrum of nanocomposite imply the synthesis of both structures together.

Evidently, the achieved peaks at 2θ = 25.76°, 30.21°, 36.58°, 45.35°, 48.31°, 52.98°, 60.16°, 62.66°, 66.99°, 76.10°, 80.43° are in accordance with the (022), (113), (004), (224), (115), (044), (026), (335), (444), (137), (008) Brag reflections (JCPDS: [00-042-1450]) demonstrating that carrollite has been synthesized with cubic crystal system in the absence and presence of FCMTs. It can be seen that FCMTs have an amorphous crystal structure, the peak at 2θ = 27.43° (d-spacing = 3.25 Å) corresponds to the (002) crystal plane indexed to the natural graphite structure^70–72^. Crystallite size of CuCo_2_S_4_ nanoparticles was 32.7 nm meanwhile it was 35.0 nm in the nanocomposite, given by Scherrer equation using (113) Brag reflection.

## Results and discussions

### FE-SEM and TEM images

**Figure 3:**
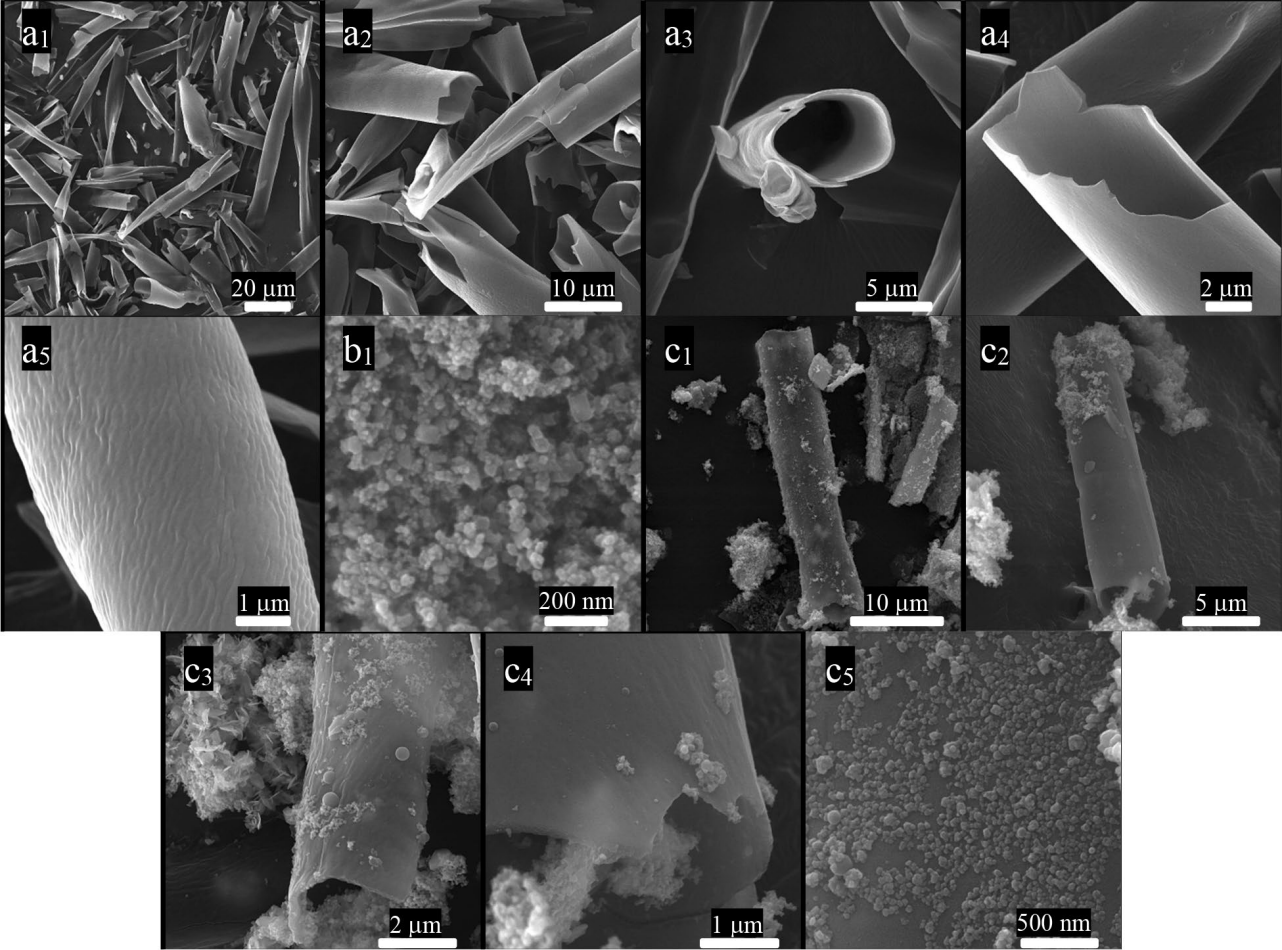
FE-SEM micrographs of FCMT (**a**_**1–5**_), CuCo_2_S_4_ (**b**_**1**_), and FCMT/CuCo_2_S_4_ (**c**_**1–5**_) structures.

**Figure 4:**
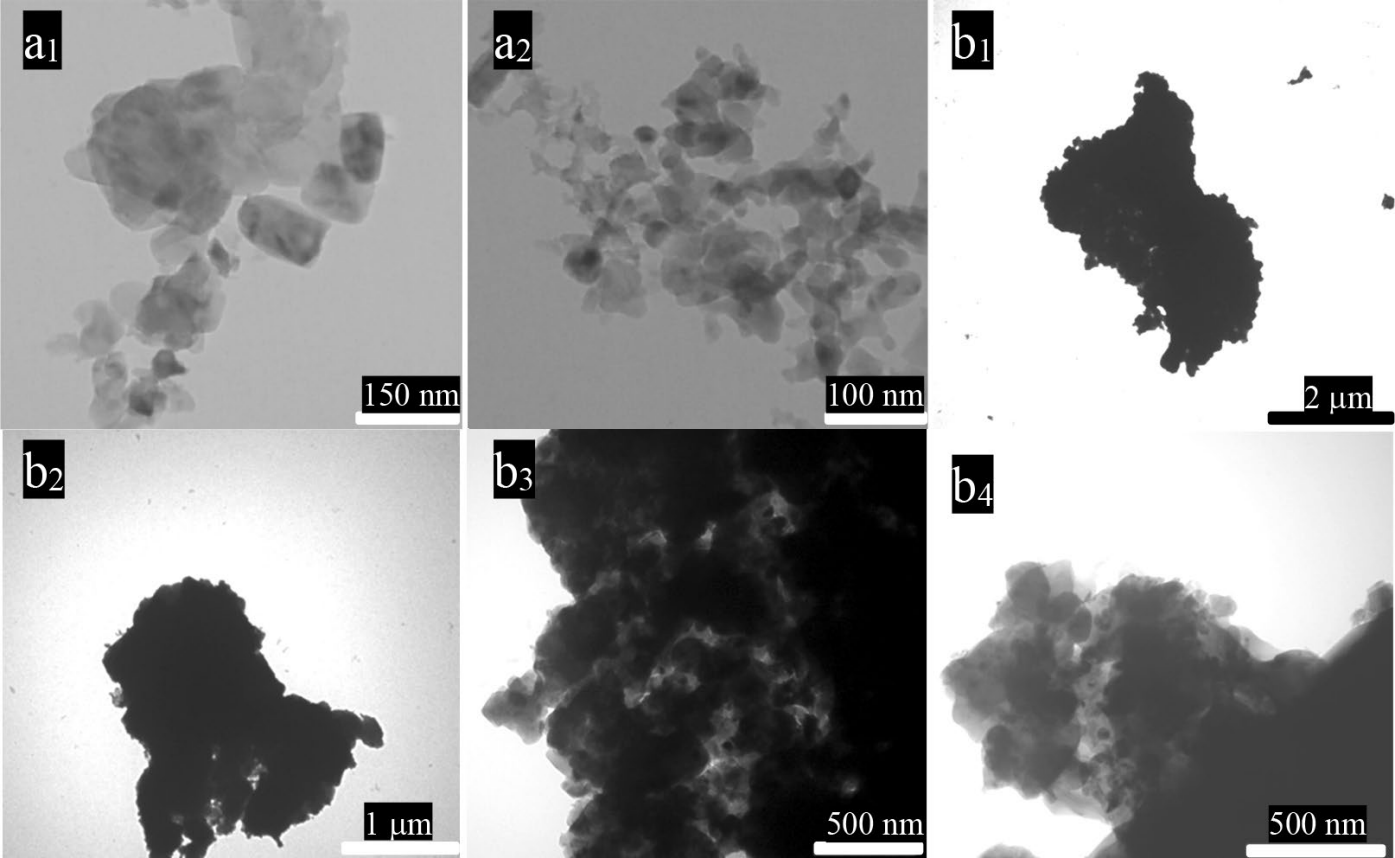
TEM images of CuCo_2_S_4_ (**a**_**1**_, **a**_**2**_) and FCMT/CuCo_2_S_4_ (**b**_**1**_–**b**_**4**_) nanostructures.

FE-SEM and TEM images of FCMT, CuCo_2_S_4_, and FCMT/CuCo_2_S_4_ structures with diverse magnifications have been exposed in Figs. 3 and 4. Obviously, FCMTs derived from the biomass have a length ranging from 15 to 60 μm with an average diameter of 5 μm and their wall thickness is below 200 nm. As revealed, the morphology of CMTs was maintained after the complementary treatments used to anchoring the functional groups onto CMTs surface. It can be seen that the uniform morphology of CuCo_2_S_4_ nanoparticles with an average thickness of 25 nm has been formed. The achieved results manifest that the novel complementary sonochemistry and solvothermal method, applied to prepare the nanocomposite, placed the nanoparticles onto the surface of FCMTs. Noticeably; the morphology of FCMTs has been maintained after the treatments.

### Optical characteristics

**Figure 5:**
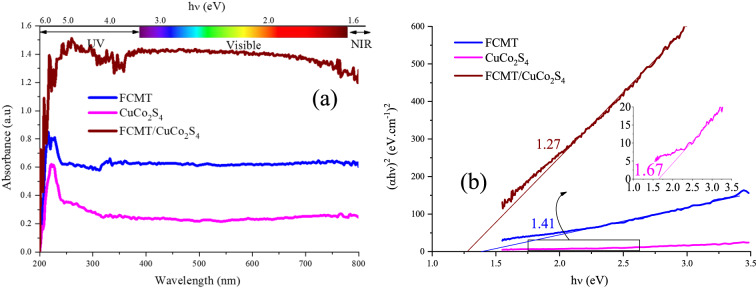
The light absorptions (λ = 200–800 nm) (**a**) and energy band gaps (**b**) of FCMT, CuCo_2_S_4_, and FCMT/CuCo_2_S_4_ structures.

Figure 5 exhibits the light absorptions (λ = 200–800 nm) and energy band gaps of FCMT, CuCo_2_S_4_, and FCMT/CuCo_2_S_4_ structures. The more polarizability is in the clear trade-off with the narrower energy band gap. Particularly, the augmenting polarizability promotes the dielectric loss in microwave absorbers. The energy band gap was defined as the distance between the valence and conduction band. As indicated, the anchoring spinel nanoparticles onto FCMTs led to the red shift of absorption edge. The following equations were used to reveal the energy band gaps: (*αhν*)^2^ = *hν − Eg*, *α* = *–1*/*t lnT*, and *T* = 10^*−*A^, where T, A, α, ν, t, h, and E_g_ are obtained by the transmittance, absorbance, absorption coefficient, frequency, thickness, Planck constant, and energy gap, given by Kubelka–Munk theory^73^. It is found that the energy band gap was diminished in the nanocomposite, realized by the produced interactions at the interfaces between FCMTs and nanoparticles as well as augmented average size of the nanoparticles reducing the distance between HOMO and LUMO, desirable for Maxwell–wagner effect and microwave attenuating^50^. The achieved results introduce the prepared nanocomposite as a promising photocatalyst.

### Magnetic properties

**Figure 6:**
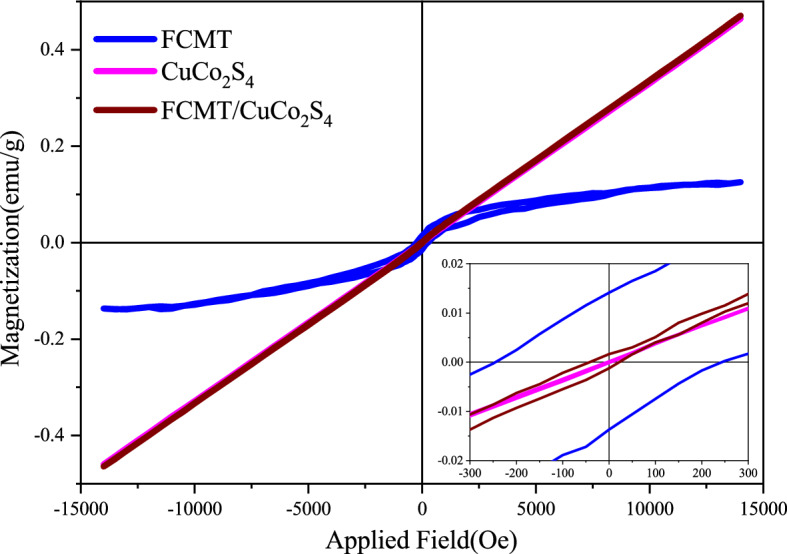
M–H loops for FCMT, CuCo_2_S_4_, and FCMT/CuCo_2_S_4_ structures.

Primitive magnetization *versus* applied field (M–H) loops for FCMT, CuCo_2_S_4_, and FCMT/CuCo_2_S_4_ structures have been illustrated in Fig. 6. It can be seen that the magnetization of CuCo_2_S_4_ and FCMT/CuCo_2_S_4_ nanostructures is augmented by enhancing the applied field. Magnetic parameters including the magnetization at an applied field of 14.5 kOe (M), remanent magnetization (M_r_), coercivity (H_c_), and isotropic M_r_/M were presented in Table 1. As revealed, H_c_ of the nanocomposite was amplified owing to the spin pinning at heterogeneous interfaces. More interestingly, intermediate structures produced by the oxygen-containing functional groups, anchored onto FCMTs, enhance the size of nanoparticles in the nanocomposite, influencing the magnetic features, defined by Snoek's law^74–76^. The observed ferromagnetic property of FCMTs is ascribed to their unique morphology as well as the produced crystal defects and distortions along the complementary oxidative treatments, hence, the presented factors induce delocalize electronic structures and develop localize dipole moments^77–80^. Natural resonance equation states that the isotropic magnetic exchange interactions and magnetization are the vital keys tuning the microwave absorbing bandwidth^81–84^.

**Table 1:** Summarized magnetic characters of FCMT, CuCo_2_S_4_, and FCMT/CuCo_2_S_4_ structures.

Entry	Sample	M (emu/g)	M_r_ (emu/g)	H_c_ (Oe)	Isotropic M_r_/M
1	FCMT	0.13	0.013	245.21	0.100
2	CuCo_2_S_4_	0.46	≈ 0.000	3.23	≈ 0.000
3	FCMT/CuCo_2_S_4_	0.47	0.002	22.09	0.004

### Antibacterial properties

**Figure 7:**
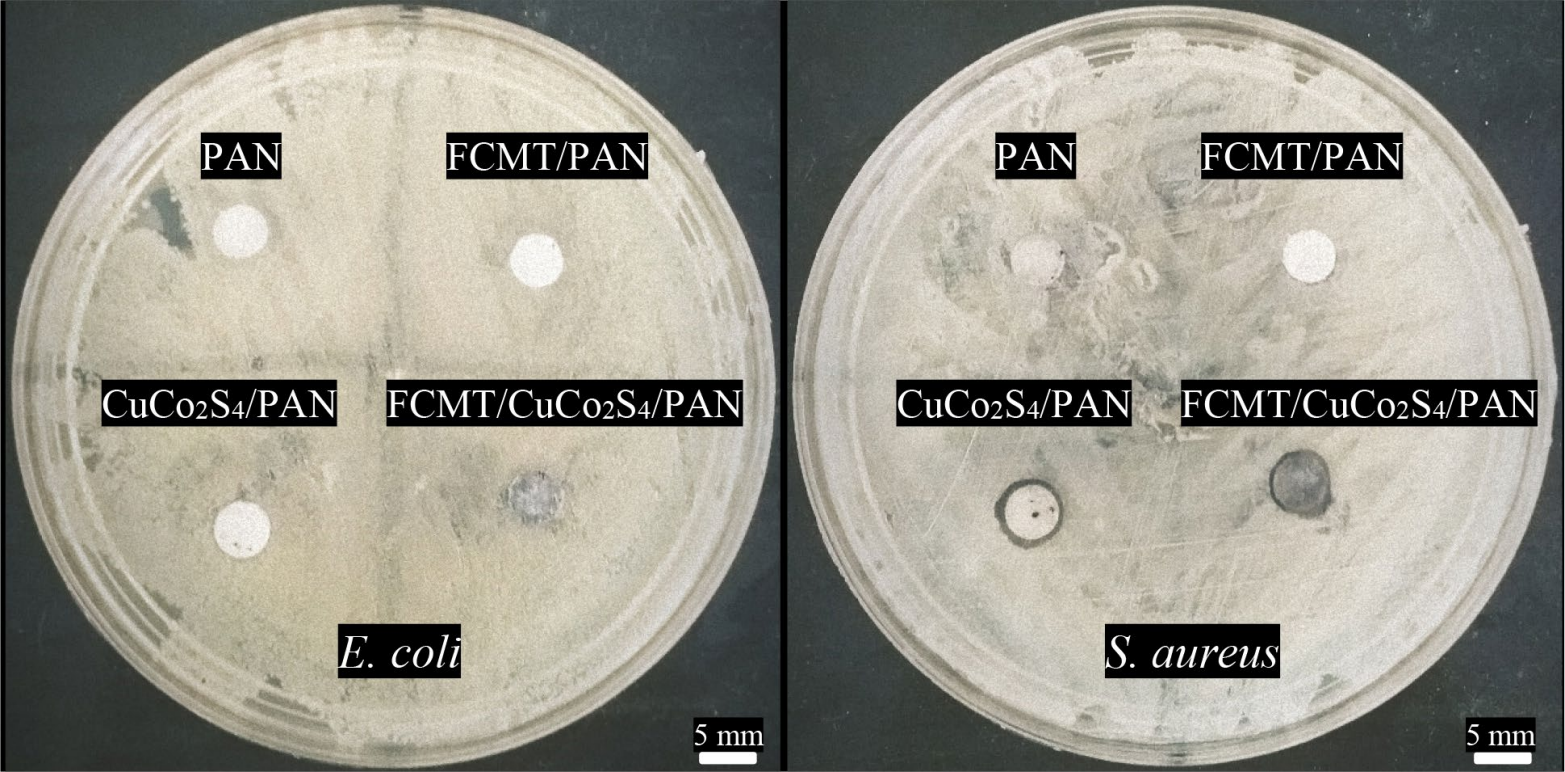
Antibacterial activity of PAN, FCMT/PAN, CuCo_2_S_4_/PAN, and FCMT/CuCo_2_S_4_/PAN against *E. coli* and *S. aureus.*

The agar diffusion method was applied to investigate the antibacterial characteristics of samples. Figure 7 and Table 2 have exposed the antibacterial activity of PAN, FCMT/PAN, CuCo_2_S_4_/PAN, and FCMT/CuCo_2_S_4_/PAN against *E. coli* and *S. aureus*. As revealed, PAN and FCMT have not any antibacterial properties against *E. coli* and *S. aureus*. However, the observed inhibition zones are derived from the antibacterial properties of CuCo_2_S_4_ nanostructures. The antibacterial characteristics of the nanostructures are essentially originated from the established reactive oxygen species (ROS) as well as released metal ions altering the structure of lipids, proteins, peptidoglycan, and DNA of bacteria, eventually destroying them^17,20,85–91^. It can be seen that the antibacterial feature of the nanoparticles has a trade-off between the type of bacteria, associated with their intrinsic structures^20,92^.

**Table 2:** Inhibition zone diameters of the samples.

Sample	*E. coli* zone of inhibition (mm)	*S. aureus* zone of inhibition (mm)
PAN	–	–
FCMT/PAN	–	–
CuCo_2_S_4_/PAN	–	6.78
FCMT/CuCo_2_S_4_/PAN	–	6.97

### Microwave absorbing and shielding characteristics

**Figure 8:**
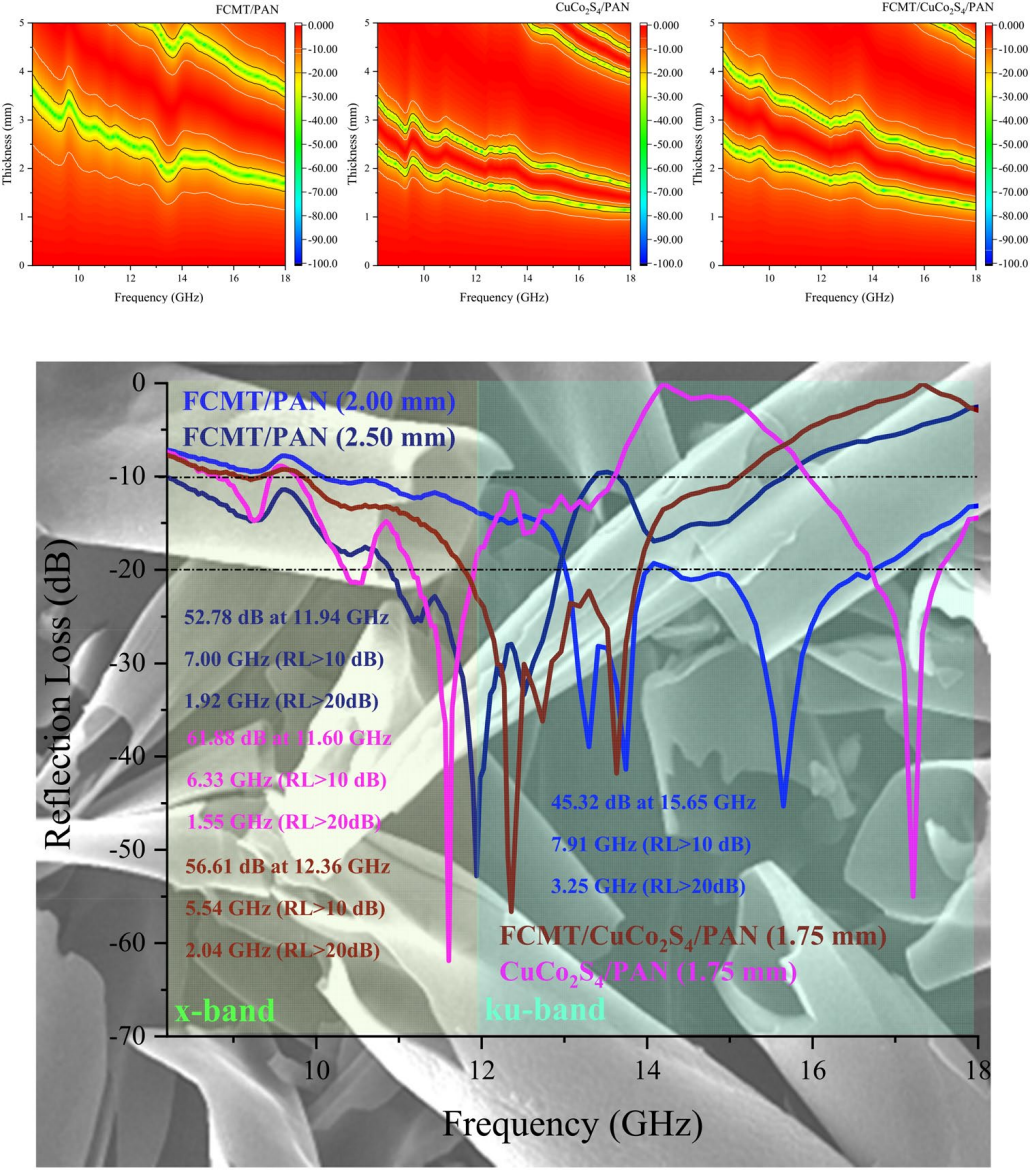
Microwave absorption and efficient bandwidth of the samples at x and ku-band frequencies.

**Figure 9:**
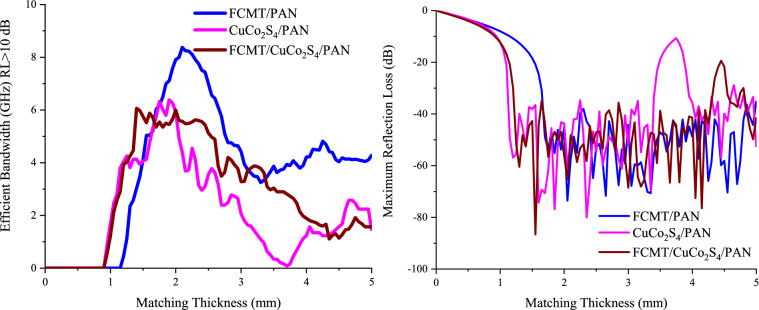
Matching thickness *versus* maximum RL and efficient bandwidth (RL > 10 dB) of the absorbers.

**Figure 10:**
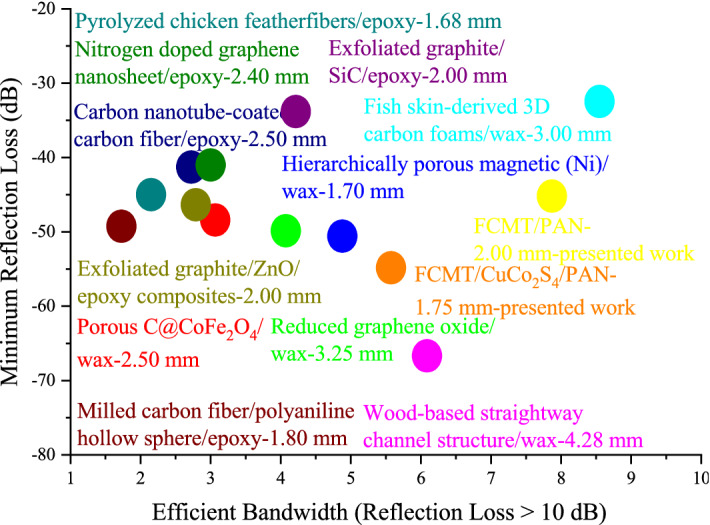
Comparing the microwave absorbing properties of the carbon-based absorbers^51–56,97–101^.

The microwave absorptions of fabricated samples were evaluated by the transmission line theory^93,94^. Figures 8 and S1 display microwave absorbing properties and simulation of matching thickness for the samples. As revealed, the maximum RL of CuCo_2_S_4_/PAN with a thickness of 1.75 mm was 61.88 dB at 11.60 GHz, while the architected FCMT/PAN composite gained a broad efficient bandwidth as wide as 7.91 GHz (RL > 10 dB) and 3.25 GHz (RL > 20 dB) with a thickness of 2.00 mm. More significantly, FCMT/CuCo_2_S_4_/PAN demonstrated a maximum RL of 56.61 dB at 12.36 GHz and an efficient bandwidth of 2.04 GHz (RL > 20 dB) with only 1.75 mm in thickness. The quarter wavelength mechanism denotes that there is a clear trade-off between the matching frequency and thickness, tuned by the relative complex permeability and permittivity^95,96^. Accordingly, CuCo_2_S_4_/PAN and FCMT/CuCo_2_S_4_/PAN composites demonstrated the thinner matching thicknesses, compared to FCMT/PAN composite. Figure 9 depicts the matching thickness *versus* maximum RL and efficient bandwidth (RL > 10 dB) of the absorbers, as well as Fig. 10 represents a comparative diagram related to the maximum RL and efficient bandwidth (RL > 10 dB) of the reported results and this research^51–56,97–101^.

**Figure 11:**
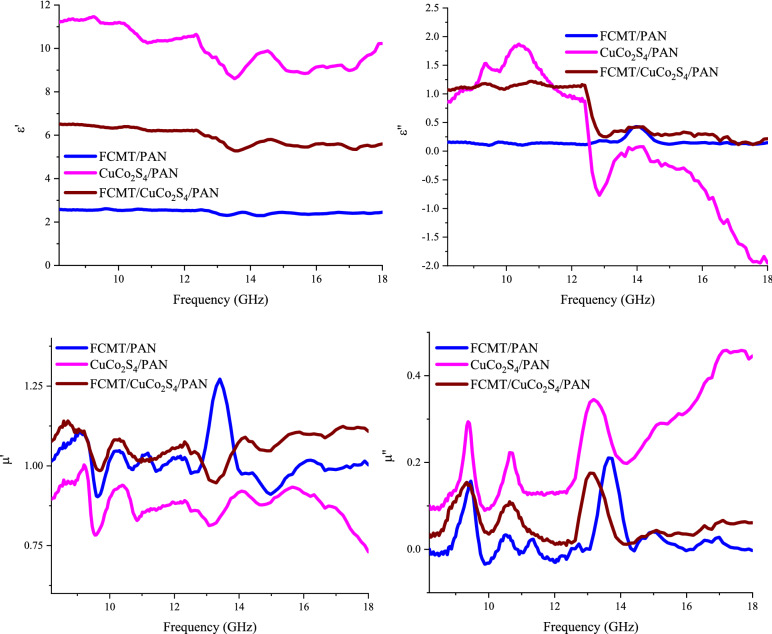
Relative complex permeability and permittivity of the absorbers from 8.2 to 18 GHz.

Frequency dependence of complex permittivity and permeability of the samples has been depicted in Fig. 11. The real part of permittivity is originated from dipole and interfacial polarizations^53,54,102^. It can be seen that FCMTs, ornamented by CuCo_2_S_4_ nanoparticles, indicated the augmented relaxation loss mechanism due to the intrinsic characteristics of FCMTs, nanoparticles, and PAN, as well as, the emerged exclusive interactions at heterogeneous interfaces. Conductive loss is the key factor, boosting the imaginary part of permittivity^53,103^. As indicated, anchoring the nanoparticles onto FCMTs amplifies the conductive loss mechanism, compared to the FCMT/PAN composite. The observed notches at permeability curves are generated from the natural and exchange resonances^82,103^. It is found that CuCo_2_S_4_ nanoparticles and FCMT/CuCo_2_S_4_ nanocomposite showed the considerable imaginary part of permeability, derived from the intrinsic features of nanoparticles. These phenomena are realized by the produced crystal defects, distortions, and dislocations, as well as the induced magnetic dipole moments, established by the unique interactions at grain boundaries^104,105^. Eddy current loss plays a vital role in microwave absorption. The more constant eddy current curve imply to the more eddy current loss mechanism^27^. Evidently, the mechanism of eddy current loss commands in the absorbing media of FCMT/CuCo_2_S_4_/PAN and FCMT/PAN composites over 14.50 GHz (Figure S2).

**Figure 12:**
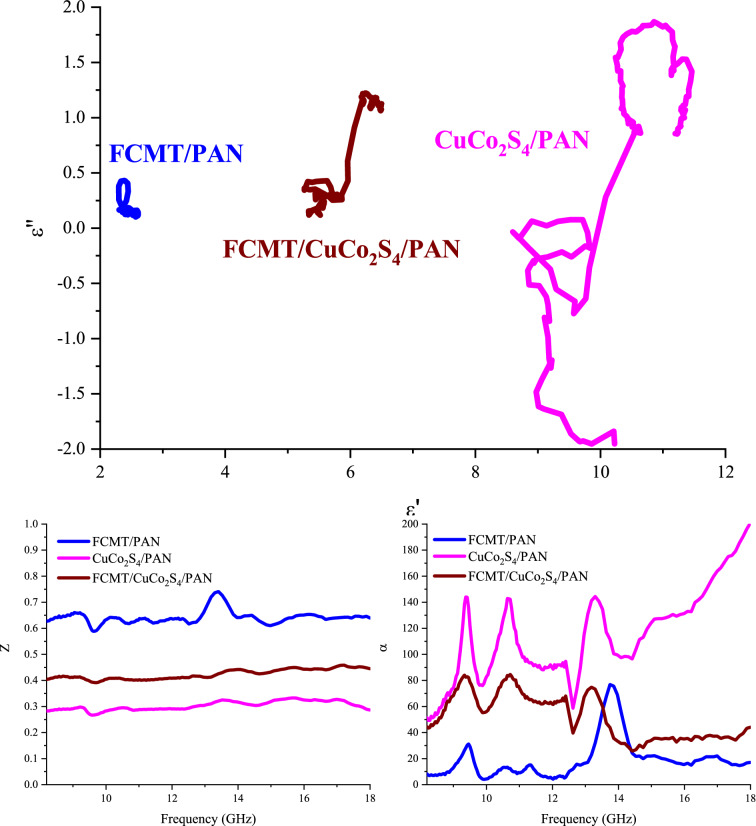
Cole–Cole plot, Z, and α for the tailored structures.

**Figure 13:**
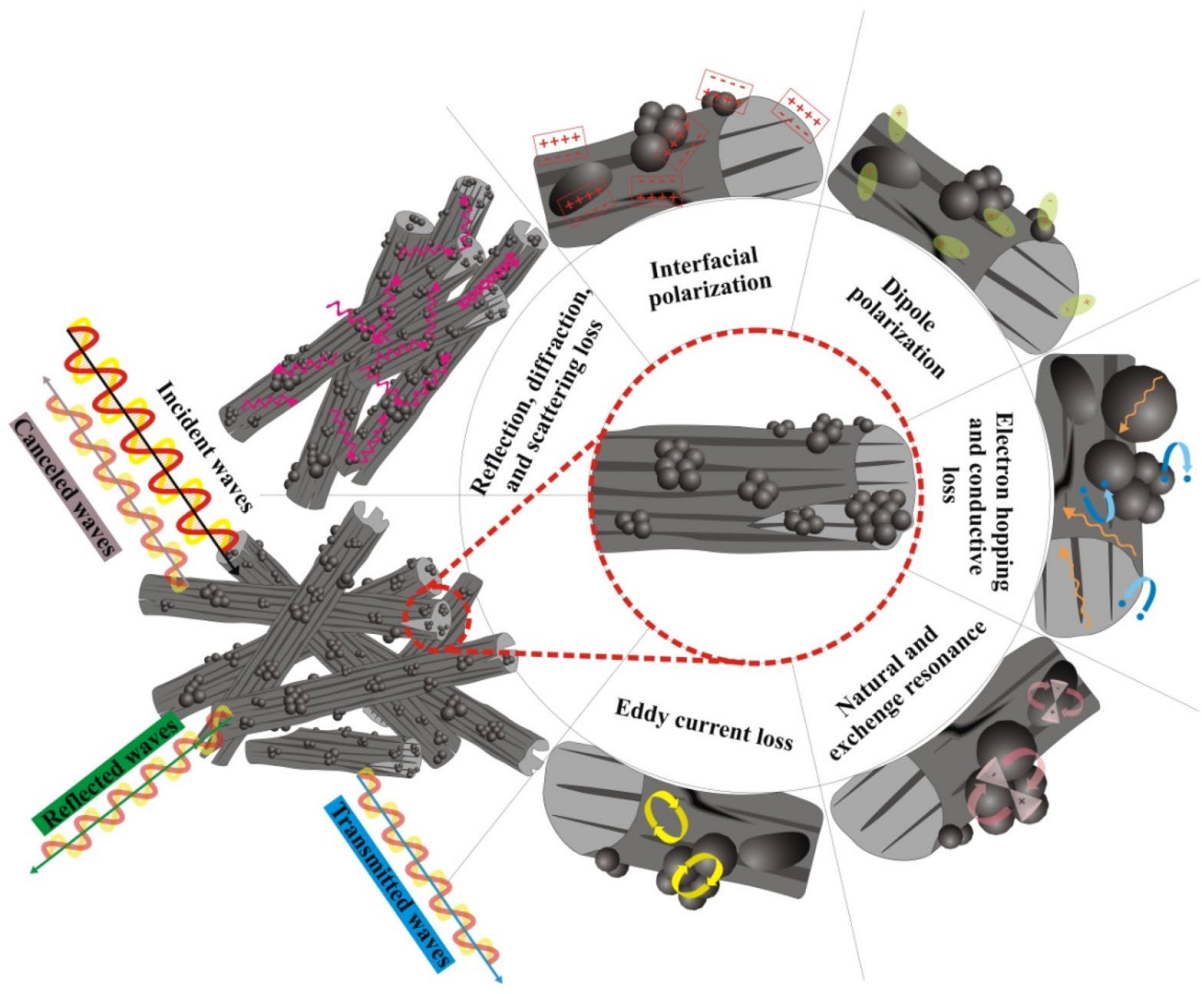
Schematic illustration of the microwave absorbing mechanisms.

Figure 12 exposes Cole–Cole plot, impedance matching (Z), and attenuation constant (α) of the samples. Cole–Cole plot is produced by drawing ε′ *versus* ε″. Each emerged semicircle denotes one relaxation loss procedure, deduced by Debye relaxation theory^106^. As given by the plot, the semicircles exhibited that the relaxation mechanism in the composites are ordered as FCMT/PAN < CuCo_2_S_4_/PAN < FCMT/CuCo_2_S_4_/PAN. Noteworthy, PAN as a novel absorbing matrix develops the dielectric characteristics of samples due to its functional groups. Z mechanism (Z = 1) is in compromise with the propagation of incident waves in the absorbing matrix^107–109^. The achieved results attest that this mechanism is the crucial factor leading to the outstanding microwave attenuation of FCMT/PAN composite. Accordingly, incident waves more influence into the absorbing medium, then absorbing mechanisms such as multiple reflections and scattering as well as the quarter wavelength and canceled waves can be elevated. α and dissipation factor (tan δ- Figure S2) estimate the ability of an absorber for energy conversion^51,100,110^. The achieved results manifest that the more α and |tan δ| are realized by the more imaginary part of permeability and permittivity. Schematic illustration of the microwave absorbing mechanisms has been shown in Fig. 13.

**Figure 14:**
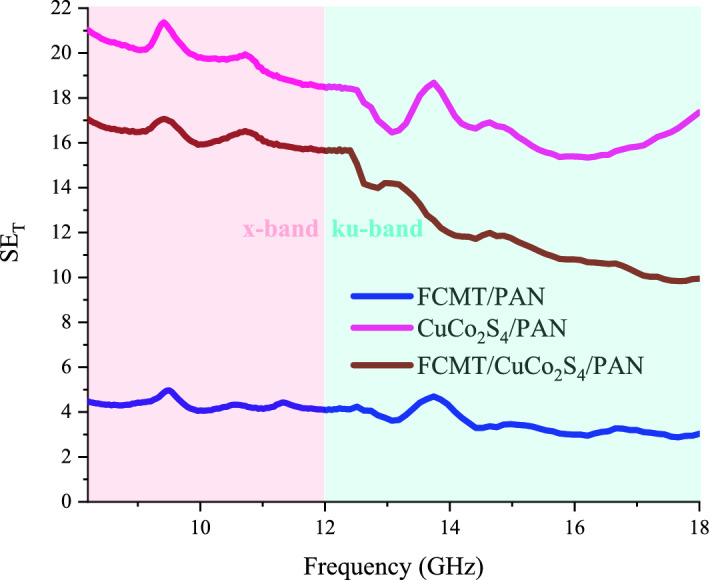
SE_T_ of the absorbers at x and ku-band frequencies.

The electromagnetic interference SEs of the samples with 2.00 mm in thickness were assessed. As known, SE_T_ is the sum of SEs attributed to the reflectance (SE_R_) and absorbance (SE_A_)^111^. Figures 14 and S3 exhibit the electromagnetic interference SEs of the samples. Interestingly, FCMT/CuCo_2_S_4_/PAN and CuCo_2_S_4_/PAN nanocomposites demonstrated more than 90 and 97% SE_T_ at entire x and ku-band frequencies. It should be noted that the eye-catching SE_T_ of the samples are derived from the absorbance, generated from the dominant microwave absorbing mechanisms existing in their absorbing medium. The achieved results testified that FCMTs as a novel carbon-based material, derived from biomass material, demonstrated outstanding microwave characteristics meanwhile anchoring the nanoparticles onto their structure promoted microwave absorbing features. More significantly, the tailored composites based on PAN, as a practical absorbing matrix, demonstrated the salient microwave absorbing properties as well as considerable SE_T_. The obtained results clarified that the remarkable microwave features of the samples are essentially generated from the dipole, interfacial, and defect polarization, conductive loss, natural and exchange resonance, eddy current loss, multiple reflections and scattering, impedance matching, as well as quarter wavelength mechanism. Applied equations to investigate microwave absorbing and shielding properties of the samples were arranged in Supplementary materials.

## Conclusion

In this research, a novel morphology of conjugated carbonaceous structures was fabricated using a novel precursor. The prepared CMTs were functionalized based on the solvothermal and sonochemical routes. Moreover, CuCo_2_S_4_ nanoparticles were tailored using a solvothermal method and anchored onto FCMTs by an innovative process, as novel microwave absorbing and antibacterial material. All of the analyses revealed that all of the samples were fabricated in good order. Noticeably, PAN was applied as a novel absorbing medium to evaluate the microwave absorbing properties of samples, demonstrating the outstanding microwave features. More significantly, microwave absorbing features and electromagnetic interference SEs of the architected samples were scrupulously dissected, illustrating that the relaxation and conductive loss, natural and exchange resonance, as well as quarter wavelength and eddy current loss are the pioneer mechanisms paving the way for the obtained salient microwave characteristics.

## Supplementary information

Supplementary Informations.
