# Supplementary material for: Architecting functionalized carbon microtube/carrollite nanocomposite demonstrating significant microwave characteristics
Source: Sci Rep. 2021 Jun 7;11:11932. doi: 10.1038/s41598-021-91370-5 (PMC8184785; doi:10.1038/s41598-021-91370-5)
Supplement: Supplementary file 1 — Supplementary Informations. [file 41598_2021_91370_MOESM1_ESM.docx]

**Supporting Information**

**Architecting functionalized carbon microtube/carrollite nanocomposite demonstrating significant microwave characteristics**

Reza Peymanfar ^*a, b^, Elnaz Selseleh-Zakerin ^b^, Ali Ahmadi ^b^, Seyed Hassan Tavassoli ^*a^

*^a^ Laser and Plasma Research Institute, Shahid Beheshti University, G. C., Evin, Tehran, 19839, Iran*

*^b^ Department of Chemical Engineering, Energy Institute of Higher Education, Saveh, Iran*

**1. Characterization**

**1. 1. Microwave absorbing features**

**Fig. S1.** Microwave absorption and simulation of matching thickness for the samples at 8.2-18 GHz

**Fig. S2.** tan δ and C_0_ for the specimens along x and ku-band frequency

**Fig. S3.** SE_A_ and SE_R_ of the absorbers at x and ku-band frequency

**Table. S1.** Applied equations to investigate microwave absorbing and shielding properties

| **Title:** | **Equation/s:** |
| --- | --- |
| Transmission line theory | $R\left( \mathrm{dB} \right)=20Log\left\vert\frac{Z_{\mathrm{in}}-Z_{0}}{Z_{\mathrm{in}}+Z_{0}} \right\vert$, $Z_{\mathrm{in}}=\sqrt{\frac{\mu_{r}}{\varepsilon_{r}}}\tanh\left[ j\sqrt{\mu_{r}\varepsilon_{r}}f\left( \frac{2\pi}{c} \right)d \right]$,$Z_{0}=\sqrt{\frac{\mu_{0}}{\varepsilon_{0}}}$, $\varepsilon_{r}=\varepsilon'-j\varepsilon''$, and $\mu_{r}=\mu'-j\mu''$ |
| Quarter wavelength mechanism | $t_{m}=\frac{\mathrm{nc}}{4f_{m}\sqrt{\left\vert\varepsilon_{r} \right\vert\left\vert\mu_{r} \right\vert}}$ |
| Eddy current loss | $C_{0}={\mu''\left( \mu^{'} \right)}^{-2}f^{-1}$ |
| Debye relaxation theory | $\left( \varepsilon^{'}-\frac{\varepsilon_{s}+\varepsilon_{\infty}}{2} \right)^{2}+({\varepsilon^{''})}^{2}=\left( \frac{\varepsilon_{s}-\varepsilon_{\infty}}{2} \right)^{2}$ |
| Impedance matching | $Z=\frac{Z_{in}}{Z_{0}}$ $=\sqrt{\frac{\mu_{r}}{\varepsilon_{r}}}$ |
| Attenuation constant | $\alpha=\sqrt{\sqrt{{(\varepsilon_{r}''{\mu_{r}}^{''}-\varepsilon_{r}'{\mu_{r}}^{'})}^{2}+{(\varepsilon_{r}'{\mu_{r}}^{''}+\varepsilon_{r}''{\mu_{r}}^{'})}^{2}}+(\varepsilon_{r}''{\mu_{r}}^{''}-\varepsilon_{r}'{\mu_{r}}^{'})}\frac{\sqrt{2}f\pi}{c}$ |
| Dissipation factor | $\tan\delta=\frac{{\delta_{r}}^{''}}{{\delta_{r}}^{'}}$ , ${\delta_{r}}^{'}=\varepsilon_{r}'{\mu_{r}}^{'}-\varepsilon_{r}''{\mu_{r}}^{''}$, and ${\delta_{r}}^{''}=\varepsilon_{r}'{\mu_{r}}^{''}+\varepsilon_{r}''{\mu_{r}}^{'}$ |
| Electromagnetic interference SE | SE: $\mathrm{SE}_{T}=\mathrm{SE}_{A}+\mathrm{SE}_{R}$, $\mathrm{SE}_{T}=-10\log\left\vert S_{12} \right\vert^{2}$,$\mathrm{SE}_{A}=-10\log(\frac{\left\vert S_{21} \right\vert^{2}}{{1-\left\vert S_{11} \right\vert}^{2}})$, and $\mathrm{SE}_{R}=-10\log{(1-\left\vert S_{11} \right\vert}^{2})$ |

**Table. S2.** Definitions of the used symbols for the microwave absorbing and shielding mechanisms [^1-10^](#_ENREF_1)

| **Symbol:** | **Definition:** | **Symbol:** | **Definition:** | **Symbol:** | **Definition:** |
| --- | --- | --- | --- | --- | --- |
| d | Thickness of absorber | c | Velocity of light in free space | Z_in_ | Input impedance |
| Z_0_ | Free space impedance | f | Frequency | n | Odd number |
| μ′ | Real part of permeability | μ″ | Imaginary part of permeability | t_m_ | Matching thickness |
| ε′ | Real part of permittivity | ε″ | Imaginary part of permittivity | f_m_ | Matching frequency |
| ε_∞_ | Permittivity at the infinite frequency | ε_s_ | Static permittivity |  |  |

**References**

1 Zhang, S., Cao, Q., Zhang, M. & Shi, X. Effects of Sr2+ or Sm3+ doping on electromagnetic and microwave absorption performance of LaMnO3. *Journal of Applied Physics* **113**, 074903 (2013).

2 Qin, F. & Brosseau, C. A review and analysis of microwave absorption in polymer composites filled with carbonaceous particles. *Journal of applied physics* **111**, 4 (2012).

3 Zhang, X.-J. *et al.* Enhanced microwave absorption property of reduced graphene oxide (RGO)-MnFe2O4 nanocomposites and polyvinylidene fluoride. *ACS applied materials & interfaces* **6**, 7471-7478 (2014).

4 Moitra, D. *et al.* Synthesis and Microwave Absorption Properties of BiFeO3 Nanowire-RGO Nanocomposite and First-Principles Calculations for Insight of Electromagnetic Properties and Electronic Structures. *The Journal of Physical Chemistry C* **121**, 21290-21304 (2017).

5 Du, M. *et al.* Design of efficient microwave absorbers based on multi-layered polyaniline nanofibers and polyaniline nanofibers/Li0. 35Zn0. 3Fe2. 35O4 nanocomposite. *Synthetic Metals* **223**, 49-57 (2017).

6 Shu, R. *et al.* Facile preparation and microwave absorption properties of RGO/MWCNTs/ZnFe2O4 hybrid nanocomposites. *Journal of Alloys and Compounds* **743**, 163-174 (2018).

7 Almasi-Kashi, M., Mokarian, M. H. & Alikhanzadeh-Arani, S. Improvement of the microwave absorption properties in FeNi/PANI nanocomposites fabricated with different structures. *Journal of Alloys and Compounds* **742**, 413-420 (2018).

8 Bora, P. J., Azeem, I., Vinoy, K., Ramamurthy, P. C. & Madras, G. Morphology controllable microwave absorption property of polyvinylbutyral (PVB)-MnO2 nanocomposites. *Composites Part B: Engineering* **132**, 188-196 (2018).

9 Wang, Y. *et al.* Fabrication and enhanced electromagnetic wave absorption properties of sandwich-like graphene@ NiO@ PANI decorated with Ag particles. *Synthetic Metals* **229**, 82-88 (2017).

10 Weir, W. B. Automatic measurement of complex dielectric constant and permeability at microwave frequencies. *Proceedings of the IEEE* **62**, 33-36 (1974).
